# Pro-inflammatory mediators and neutrophils are increased in synovial fluid from heifers with acute ruminal acidosis

**DOI:** 10.1186/s12917-019-1974-x

**Published:** 2019-07-03

**Authors:** Alejandra I. Hidalgo, María D. Carretta, Pablo Alarcón, Carolina Manosalva, Ananda Müller, Max Navarro, María A. Hidalgo, Thilo Kaehne, Anja Taubert, Carlos R. Hermosilla, Rafael A. Burgos

**Affiliations:** 1 Laboratory of Inflammation Pharmacology, Faculty of Veterinary Sciences, Institute of Pharmacology and Morphophysiology, Universidad Austral de Chile, Valdivia, Chile; 2 Faculty of Sciences, Institute of Pharmacy, Universidad Austral de Chile, Valdivia, Chile; 3 Veterinary Clinical Sciences Institute, Faculty of Veterinary Sciences, Universidad Austral de Chile, Valdivia, Chile; 4 Institute of Experimental Internal Medicine, Otto-von-Guericke University Magdeburg, Leipziger Strasse 44-0, 39120 Magdeburg, Germany; 5 Institute of Parasitology, Faculty of Veterinary Medicine, Justus Liebig University Giessen, 35392 Giessen, Germany

**Keywords:** Acute ruminal acidosis, Synovial fluid, Synovitis, Cytokine, Neutrophil extracellular traps, Prostaglandin E_2_

## Abstract

**Background:**

Acute ruminal acidosis (ARA) is a metabolic disease of cattle characterized by an aseptic synovitis. ARA is the result of an increased intake of highly fermentable carbohydrates that frequently occurs in dairy cattle subjected to high production requirements. In human joint diseases such as rheumatoid arthritis and gout, several pro-inflammatory molecules are increased in the synovial fluid, including cytokines, prostaglandin E_2_ (PGE_2_), metalloproteinases, and neutrophil extracellular traps (NETs). The aim of this study was to identify the presence of proinflammatory mediators and neutrophils in the synovial fluid of heifers with ARA, induced by an oligofructose overload.

Five heifers were challenged with an oligofructose overload (13 g/kg BW) dissolved in water. As a control, a similar vehicle volume was used in four heifers. Synovial fluid samples were collected from the tarso-crural joint and PGE_2_, IL-6, IL-1β, ATP, lactate dehydrogenase (LDH), albumin, glucose, matrix metalloproteinase-9 (MMP-9), cellular free DNA, NETs, and serpin B1 were analyzed at 0, 9, and 24 h post treatment.

**Results:**

At 9 h post oligofructose overload, an increase of IL-1β, IL-6, PGE_2_, serpin B1 and LDH was detected in the joints when compared to the control group. At 24 h, the synovial fluid was yellowish, viscous, turbid, and contained abundant neutrophils. An increase of DNA-backbone-like traps, histone 3 (H_3_cit), aggregated neutrophil extracellular traps (*agg*NETs), and serpin B1 were observed 24 h post treatment. Furthermore, albumins, LDH, ATP, MMP-9, IL-6, and IL-1β were increased after 24 h.

**Conclusions:**

The overall results indicate that IL-1β, IL-6 and PGE_2_, were the earliest proinflammatory parameters that increased in the synovial fluid of animals with ARA. Furthermore, the most sever inflammatory response in the joint was observed after 24 h and could be associated with a massive presence of neutrophils and release of *agg*NETs.

**Electronic supplementary material:**

The online version of this article (10.1186/s12917-019-1974-x) contains supplementary material, which is available to authorized users.

## Background

Acute ruminal acidosis (ARA) is a metabolic-nutritional disease that affects production efficiency, mainly in dairy cattle [1, 2]. A high intake of non-structural carbohydrates, which are fermented in the rumen reduce the pH of the ruminal fluid causing metabolic acidosis [3]. Lameness is frequently associated with this metabolic imbalance, and the appearance of laminitis [1] and synovitis [4]. In addition, some evidence suggests the existence of a link between the disruption in gastrointestinal functionality and joint inflammation in ruminants. Acute, sterile arthritis and tenosynovitis in cattle has previously been associated with rumen acidosis (Dirksen, 2002). Hyldgaard-Jensen and Simesen (1966) observed acute, bilateral, serofibrinous arthritis in the tarsal joints after experimental induction of ruminal acidosis in one cow. Other authors reported polysynovitis in two calves and one cow with experimentally-induced ruminal acidosis with or without endotoxin exposure [5, 6]. In spite of this, the findings were not documented and either the presence of pro-inflammatory markers in the joint of cattle with ARA, has not been measured yet.

In humans, high levels of pro-inflammatory cytokines and prostaglandin E_2_ (PGE_2_) have been found in the synovial fluid of patients with osteoarthritis [7], rheumatoid arthritis (RA) [8, 9] and gout [10, 11]. The fibroblast-like synoviocytes of RA patients trigger intracellular signaling pathways that control PGE_2_ and pro-inflammatory cytokine production e. g. interleukin 6 (IL-6) and interleukin-1beta (IL-1β), through mitogen-activated protein kinases and nuclear factor-kappa B pathways [12]. In addition, neutrophils are the main innate immune cell population present in aseptic inflammatory joint diseases [13, 14], contributing to the release of matrix metalloproteinase-9 (MMP-9) [15], pro-inflammatory cytokines [13], PGE_2_ [16], and neutrophil extracellular traps (NETs) [17, 18].

The effects of ARA on the inflammatory process in bovine joints have scarcely been studied. Some evidence suggests that heifers with ARA induced by oligofructose overload presented lameness characterized by an increased joint distention score [19] and aseptic synovitis [4]. Additionally, D-lactate can induce the release of MMP9 in vitro [20] and trigger the adhesion of bovine neutrophils to vascular endothelium through a mechanism that is dependent on the formation of NETs [21]. These antecedents suggest that during ARA there is an increase of pro-inflammatory agents and neutrophil recruitment in the synovium.

We hypothesize that in heifer with ARA induced by oligofructose overload, an increase of pro-inflammatory mediators and a large presence of neutrophils in the joint would contribute to explain the early onset of synovitis in cattle.

## Methods

### Animals and housing

Nine non-pregnant black Friesian dairy heifers aged 10–18 months and weighing 200–250 kg were used in this study. The animals were obtained from *Estación Experimental Agropecuaria Austral* farm of the Universidad Austral de Chile and were housed in the large animal facility of the Veterinary Hospital of the Universidad Austral de Chile. The health status of the animals was verified with a clinical examination and complementary tests (hematological and biochemical analysis). In addition, the animals were free of brucellosis, leucosis, and tuberculosis, and were certified by the National Livestock Service of Chile. The animals were submitted to a 4-week period of acclimatization before the experiments were conducted and carefully handled to avoid inducing stress throughout experiment settings.

The heifers were fed twice daily. The daily ration of concentrate was equally divided into two meals of 1.0 kg each of Cosetan® (IANSAGRO S.A., Chile), and water ad libitum. The heifers grazed on naturalized pasture composed primarily of perennial grasses, mostly *Holcus lanatus* and *Agrostis capillaris*. The contribution of forage legumes was low, < 10% of the dry matter. During the challenge heifers were fed individually twice a day and the meals were weighed to estimate consumption.

### Oligofructose overload

The animals were randomly assigned into two groups, oligofructose and control. For each heifer (*n* = 5), 13 g/kg of body weight (BW) of oligofructose (Orafti P95, Beneo-Alfa Group, Santiago, Chile) was dissolved in warm tap water and administered in a volume of 2 L/100 kg of BW as a ruminal drench. The same volume of water (vehicle) was used as the control group (*n* = 4). Prior to this, 5% of the main dose was provided twice daily for 3 days before the main overload, as previously described [22, 23]. The experiment included a 3-day control period before the oligofructose overload and a 24 h surveillance period afterwards. All animals were clinically monitored (heart and respiratory rate, rectal temperature, ruminal frequency, and for signs of lameness) by a veterinary clinician, and all procedures were performed in the ruminant unit of the Veterinary Hospital.

Twenty-four hours after the administration of the total oligofructose dose, a solution of sodium bicarbonate (1 g/kg BW) and ruminal restorative mineral salts (Bilifar®, DragPharma, Chile) were administered orally, and analgesic/anti-inflammatory (Febrectal®, DragPharma, Chile)- and antibiotic (Pencidrag®, DragPharma, Chile)-treatments were given by intramuscular injection. At the end of the study, none of animals had to be euthanized, and all fully recovered. Moreover, none of them exhibited secondary pathologies after two months of observation period. Finally, all animals returned to the farm of the Universidad Austral de Chile.

### Animal welfare standards

All experiments were conducted in accordance with the guidelines for the use of animals in experimentation of the Universidad Austral de Chile, and the National Guidelines on the Use of Experimental Animals of the ‘Comisión Nacional de Ciencia y Tecnología de Chile’. All animal experiments were also approved the Institutional Ethic Review Committee (No. Bioethics Report 217/2015 and No. bioethics monitoring S-49-2017).

### Ruminal, synovial fluid and blood sample collection

During the following 24 h after the challenge, the animals were sampled to obtain ruminal and synovial fluid. Three schedules were adopted for obtaining samples: 0 h, corresponding to samples before administration of the total dose of oligofructose; 9 h after the administration of the total dose of oligofructose, time point where an increase in plasma lactate [24] and ruminal acidosis are observed [22]; and 24 h after the administration of oligofructose overload which represented the period of maximum WBC count detected in the synovial fluid of heifers [25].

Rumenocentesis was performed in the dorsal sac of the rumen to collect ruminal fluid [26]. Afterward, the pH was quickly measured in a portable pH meter with a calibration check™ (Hanna Instruments, RI, USA) to assess the ruminal acidosis experimental procedure.

To obtain synovial fluid an aseptic arthrocentesis was performed [25, 26] using an 18 G needle of 3.8 cm for the collection of 3–15 ml of fluid. The punctures were performed randomly only once in each joint, to avoid sample contamination; the puncture sites were: between the *talus* and the *os centroquartale* of the tarsal joint and between the intermediate and lateral patellar ligaments of the knee joint.

The blood sample was collected by venipuncture of the jugular vein and plasma isolation was performed according Concha et al. (2014) and stored at − 80 °C.

### Synovial fluid characterization

For the physical characterization of the fluid, color was assessed by visual inspection. The pH was measured immediately after obtaining a sample of synovial fluid, using a portable pH meter (Hanna instruments, RI, USA).

### Cytological analysis

Cellular characterization was performed after preparing a cellular smear. For this, 30 μl of fresh synovial fluid was centrifuged at 200×*g* for 10 min in a cytospin centrifuge (Hettich, Germany). Staining was performed using a Hemacolor microscope kit (Merck®, Germany). Neutrophil count was performed by observing 5 fields with an Olympus BX51® microscope (Olympus, Japan). The results were expressed as the average of five observed fields.

### Biochemical analysis

Synovial fluid was centrifuged at 1000×*g* for 10 min at RT, and the supernatant was stored at − 80 °C. For albumin detection, the photometric-colorimetric method bromocresol green (HUMAN Diagnostic Worldwide, Germany) was used. Glucose was estimated by using glucose oxidase-phenol and the 4-aminophenazone enzymatic colorimetric method (HUMAN Diagnostic Worldwide). For lactate dehydrogenase (LDH) the kinetic method according to the Scandinavian Committee on Enzymes (HUMAN Diagnostic Worldwide) was used. All analyses were performed in a Metrolab 2300® autoanalyzer, according to the manufacturer’s instructions (Wiener Lab Group, Argentina).

### IL-1β, IL-6, and PGE_2_ measurements

Aliquots of 200 μl of synovial fluid were used to estimate the concentration of pro-inflammatory cytokines by using a bovine IL-1β ELISA Kit (#ESS0027, Thermo Fisher Scientific, MA, USA) and IL-6 (#ESS0029, Thermo Fisher Scientific, MA, USA), according to the manufacturer’s instruction. Briefly, the capture antibody was incubated overnight; wells were then blocked for 1 h; subsequently, 200 μl of sample was added and incubated for 1 h. After the plates had been washed twice, the detection antibody was added and incubated for 1 h. After further two washes, streptavidin was added and the mixture incubated for further 30 min. Finally, tetramethylbenzidine substrate solution (TMB) was added followed by incubation for 20 min in the dark. All procedures were performed at RT. The reaction was stopped with 0.16 M H_2_SO_4,_ and the samples were analyzed for IL-1β and IL-6 at 450 nm and 550 nm, respectively, in an automatic Varioskan Flash Reader (Thermo Fisher Scientific, MA, USA).

For PGE_2_ analysis an ELISA Kit–Monoclonal (#514010, Cayman Chemical, MI, USA) was used according to the manufacturer’s instructions. Briefly, in a 96 well plate, 50 μl of ELISA buffer, 50 μl of sample, 50 μl of PGE_2_ acetylcholinesterase tracer, and PGE_2_ monoclonal antibody were combined. The incubation was performed for 18 h at 4 °C. The next day, the contents of each well were washed 5 times and 200 μl of Ellman’s reagent was added, followed by agitation in the dark for 90 min. Finally, the plate was analyzed at 405 and 420 nm in a Varioskan Flash Reader (Thermo Fisher Scientific MA, USA).

### ATP measurement

A CellTiter-Glo™ luminescent cell viability assay (Promega, WI, USA) was used according to the manufacturer’s instructions. Briefly, equal volumes of synovial fluid and CellTiter-Glo™ Reagent (50 μl) were added, mixed for 4 min on an orbital shaker and then incubated at RT for 10 min. Luminescent analysis was performed in a Varioskan Flash Reader (Thermo Fisher Scientific, MA, USA).

### Zymography

Synovial fluid samples were centrifuged at 1000×*g* 10 min and the supernatant was stored at − 80 °C. MMP-9 activity was carried out by zymography [27]. Briefly, the sample was diluted 1:10 in distilled water, and 4 μl was loaded onto 10% polyacrylamide gels (0.75 mm thick) containing 0.28% gelatin. The gels were run at 200 V for 1 h in a Bio-Rad Mini Protean II apparatus (Bio-Rad Laboratories, CA, USA) and then washed thrice in 2.5% Triton X-100 in distilled water on a shaker at RT for 30 min. Subsequently, the gels were incubated in reaction buffer consisting of 100 mM Tris (pH 7.5) and 10 mM CaCl_2_ at 37 °C overnight. The gels were stained with 0.5% Coomassie Brilliant Blue R-250 (Winkler, Santiago, Chile) in a solution of acetic acid, methanol, and water (1:3:6). Gelatinase activity was determined by the observation of non-staining areas in which the gelatin was degraded. Calculation of the apparent molecular masses of the gelatinolytic bands was estimated by using a pre-stained molecular mass marker (Fermentas International Inc., Canada). Band intensity was determined using ImageJ 1.51j8 software.

### Cellular free DNA (cf-DNA)

cf-DNA was measured to evaluate NET formation [28]. For quantification of cf-DNA, 200 μl of freshly extracted synovial fluid was used. Samples were incubated with 0.5 U/ml per well of micrococcal nuclease (New England Biolabs, USA) for 30 min at 37 °C. Then samples were centrifuged at 500×*g* for 5 min at RT. Afterwards, 100 μl of supernatant was transferred to a new well and 100 μl of Quant-iT™ PicoGreen™ dye (Thermo Fisher Scientific, MA, USA) diluted in TE buffer (10 mM Tris, 1 mM EDTA) was added. Analysis was performed using an automated Varioskan Flash Reader (Thermo Fisher Scientific, MA, USA) at 484 nm excitation and 520 nm emission, as described elsewhere [29].

### Western blot

Synovial fluid (stored at − 80 °C) was used to quantify total protein using the Bradford colorimetric method [30]. For this, 50 μg of total protein were separated on 12% SDS-PAGE gels at 100 V for 2 h and transferred onto PVDF membranes at 200 mA for 2 h. Membranes were then blocked in Tris-buffered saline with Tween-20 (TBS-T) buffer (10 mM Tris–HCl, 68 mM NaCl, and 0.1% Tween-20) and 5% non-fat dry milk for 2 h at RT and washed with TBS-T. The primary antibodies used were: histone H3 (citrulline R2 + R8 + R17; H_3_cit) antibody (#ab5103, Abcam, UK) and serpin B1 antibody (#ARP52417_P050, Aviva Systems Biology, CA. USA) incubated at 4 °C in constant agitation overnight. Thereafter secondary antibodies [anti-rabbit IRDye 800 and 680 (Li-cor Biosciences, USA)] were used. Analysis of membranes was performed by using a LI-COR OdysseyFc system (LI-COR Biosciences, NE, USA) and densitometry was quantified using the Image 1.51j8 software.

### Fluorescence microscopy

Aliquots of 100 μl of fresh synovial fluid were placed on slides and fixed with 2% paraformaldehyde for 15 min at RT, then washed twice with cold PBS. Primary antibody histone H3 [citrulline R2 + R8 + R17] (H_3_cit) (#ab5103, Abcam, United Kingdom) was incubated overnight at 4 °C. The following day the secondary antibody, Alexa Fluor 594 (#A11037, Invitrogen, USA), was incubated for 2 h at RT. Finally, DNA was stained with Quant-iT™ PicoGreen™ dye (Thermo Fisher Scientific, MA, USA). Images were acquired with an OLYMPUS® Fluoview 1000 confocal microscope.

### Statistical analyses

Since our data do not show normal distribution and variance homogeneity according to Shappiro-Wilks and Bartlett’s test respectively, a Mann-Withney test for non- parametric data was used. A *P-value* < 0.05 was considered as significant. Data were depicted in bar graph as mean ± S.E.M. All analyses were performed using the GraphPad Prism® software (v 5.0, Graphpad Software, CA, USA).

## Results

### Physical changes and increase of neutrophils in the synovial fluid of heifers with ARA

**Fig. 1:**
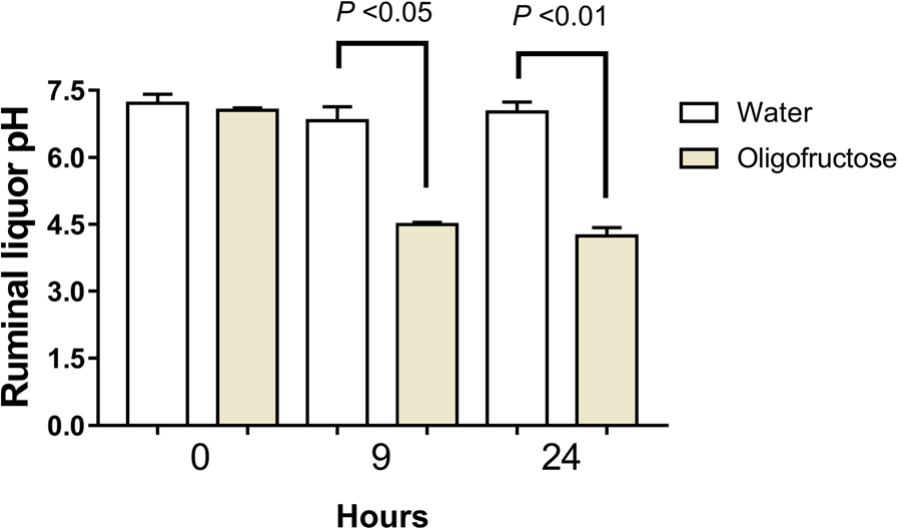
Ruminal pH at 0, 9, and 24 h post overload of oligofructose and water administration. Each bar represent mean ± SEM

To experimentally induce ARA in heifers, we used the oligofructose overload protocol [22]. A decrease of ruminal pH < 5.0 as clinical diagnostic criteria of ARA was used [31]. In fact, at 9 h and 24 h the experimental procedure significantly reduced the ruminal pH to around 4.5, confirming ARA induction (Fig. 1).

**Fig. 2:**
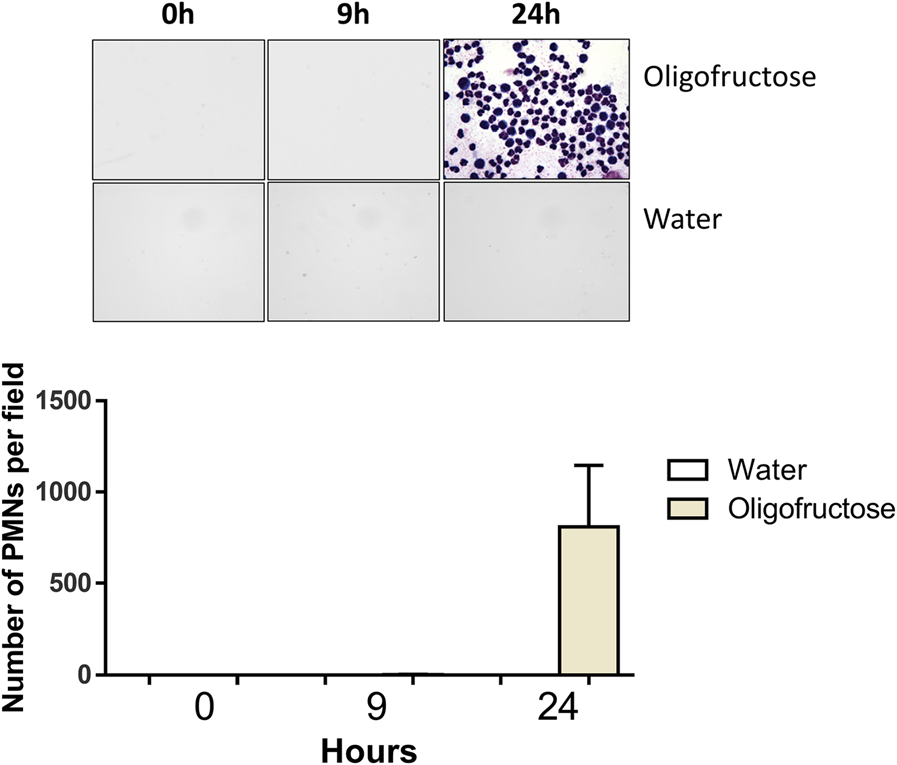
Presence of neutrophils in the synovial fluid of animals with ruminal acidosis. Smear of synovial fluid at 0, 9, and 24 h after oligofructose overload and water treatment are depicted. Each bar shows the arithmetic mean ± SEM

The synovial fluid of the animals before induction, at 0 h, was colorless, transparent, and had a fluid consistency; similar findings were observed after 9 h. At 24 h, the animals were weak, slow-moving, prone to decubitus, anorexic, and presented explosive liquid diarrhea. At 9 h and 24 h, all animals treated with oligofructose overloads showed lameness resulting in a locomotion score of 2–3 [32]. In addition, at 24 h, there was an evident physical change in synovial fluid, which became yellowish, viscous, and turbid. Additionally, after 24 h, a marked increase in neutrophil content within synovial fluid was detected (Fig. 2).

As described elsewhere [4], microbiological analysis of all synovial samples were negative (data not shown).

### IL-1β, IL-6, PGE_2_, ATP, and MMP-9 increased in the synovial fluid of heifers with ARA

**Fig. 3:**
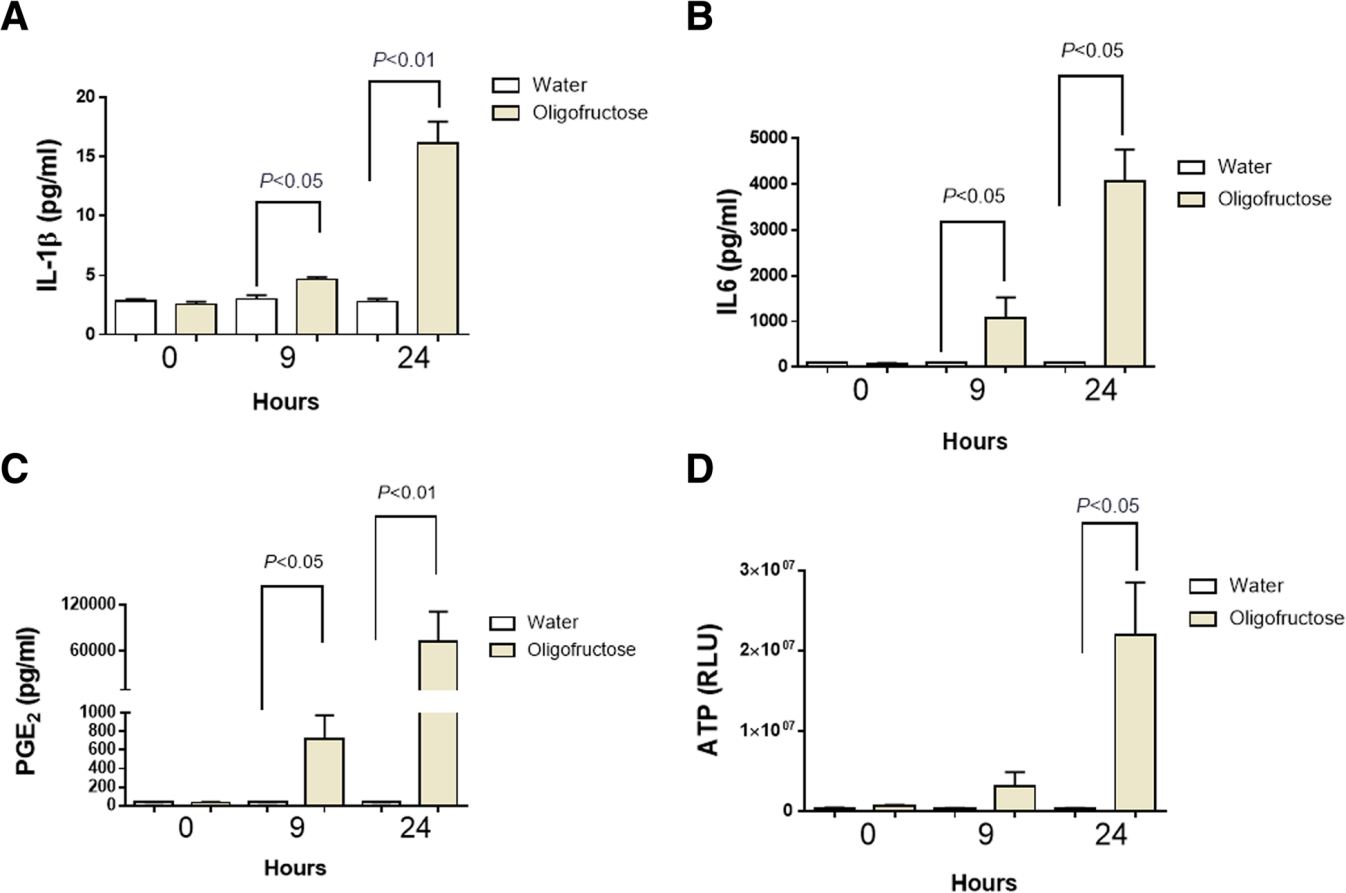
Analysis of the pro-inflammatory mediators in the synovial fluid of heifers with acute ruminal acidosis (ARA). Levels of IL-1β (**a**), IL-6 (**b**), PGE_2_ (**c**), and ATP (**d**), in synovial fluid at 0, 9, and 24 h post induction of ARA, were detected. Relative luminescence units (RLU). Each bar shows the arithmetic mean ± SEM

The concentration of several inflammatory parameters such as IL-1β, IL-6, PGE_2_, ATP and MMP-9 in the synovial fluid, were assessed in animals with ARA. At 9 h IL-1β, IL-6, PGE_2_ showed a significant increase compared to the vehicle which was more evident after 24 h (Fig. 3A-C). In plasma samples did not observe any significant changes in these proinflammatory parameters (Additional file 1).

**Fig. 4:**
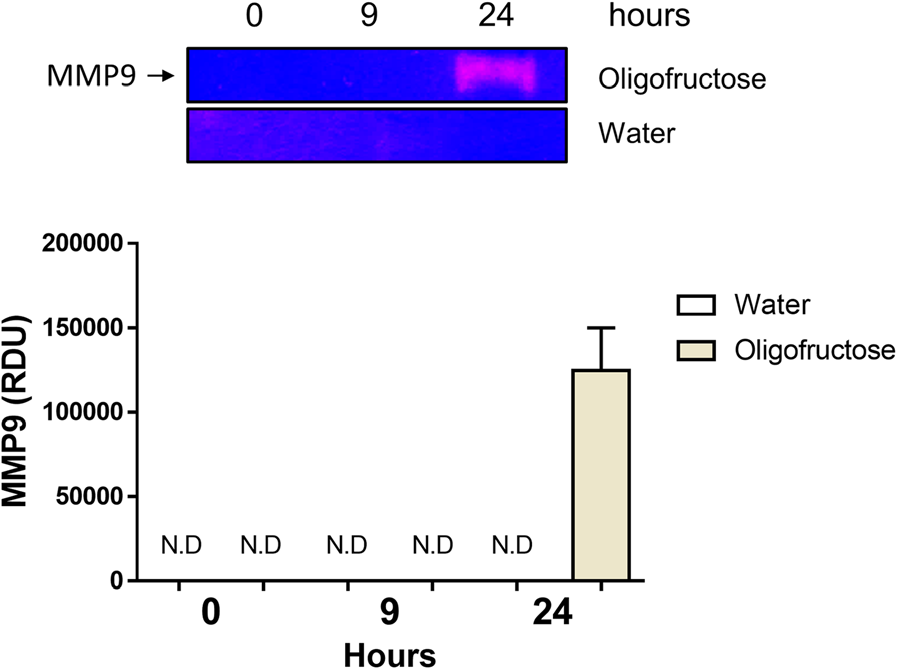
Presence of MMP-9 in the synovial fluid of heifers with ARA. A representative zymography is depicted. Each bar shows the arithmetic mean ± SEM of relative unit of densitometry (RDU). N.D. none detected

ATP is a metabolite with a pro-inflammatory role in joints [33] and in a similar fashion we observed a significant ATP increase (*P* < 0.05) at 24 h (Fig. 3D). Because neutrophil recruitment was detected at 24 h, and these cells release MMP-9 from intracellular granules, we assessed MMP-9 activity in joints using zymography. An increase of MMP-9 activity was recorded only at 24 h (Fig. 4).

### Presence of *agg*NETs, histone citrunillated 3, and serpin B1 in the synovial fluid of heifers with ARA

**Fig. 5:**
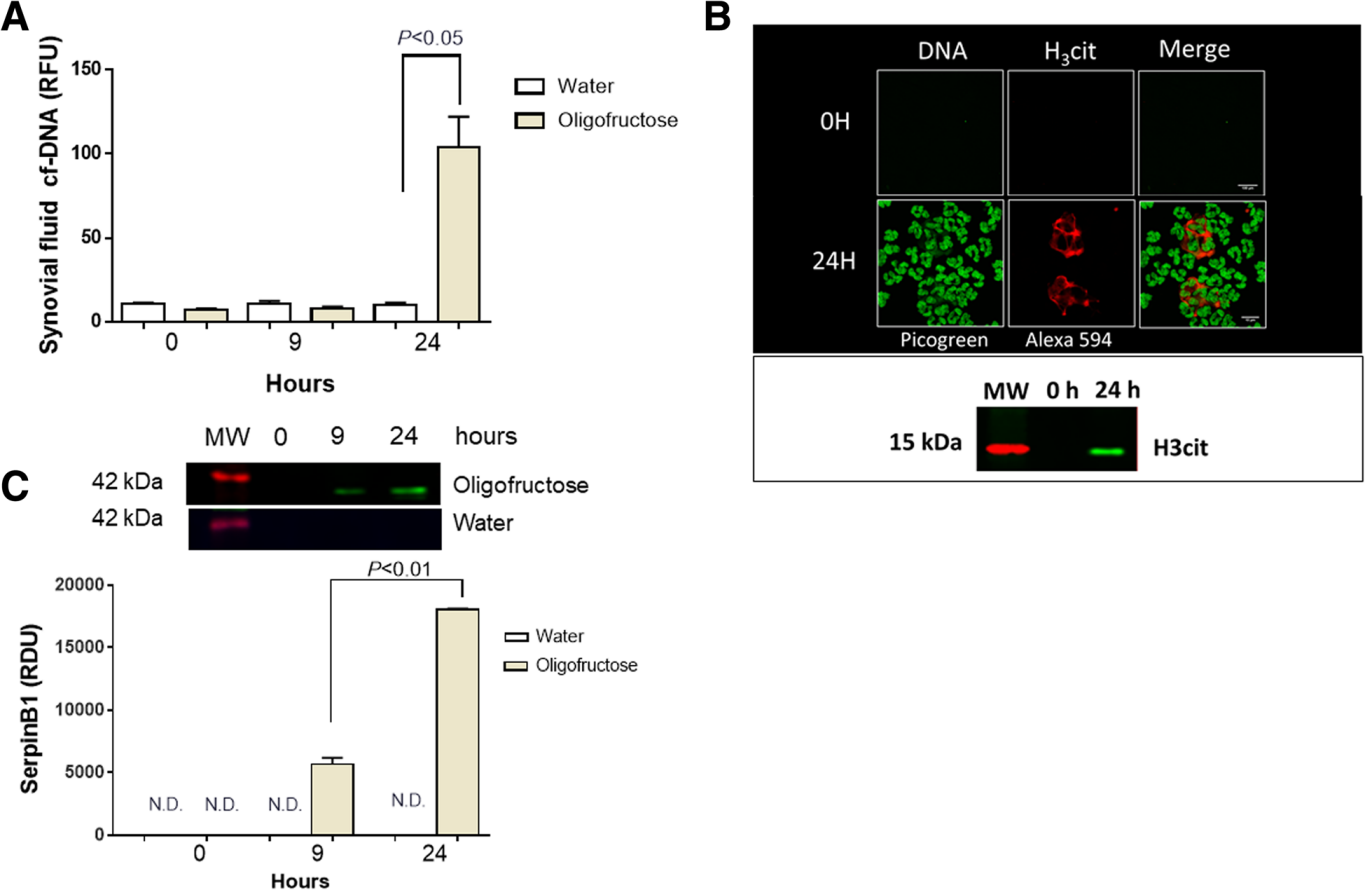
Determination of neutrophil extracellular traps (NETs) and serpin B1 in the synovial fluid of heifers with acute ruminal acidosis (ARA). **a** cf-DNA measurement at 0, 9, and 24 h. Relative fluorescence units (RFU). **b** Immunofluorescence of synovial fluid at 0 h and 24 h after the induction of ARA. Histone H3cit and DNA at 24 h post induction was observed. A representative western blot of synovial fluid to detect H_3_cit at 24 h post induction is shown. Scale bar 100 μm and 10 μm for 0 h and 24 h respectively. **c** Immunoblot of serpin B1 at 0, 9, and 24 h in the synovial fluid. A bar graph of the relative densitometry units (RDU) of serpin B1 is shown. Each bar shows the arithmetic mean ± SEM. N.D. none detected

The presence of NETs in synovial fluid is a hallmark in the joint aseptic inflammatory process [14, 34]; for this we assessed the presence of DNA traps in synovial fluid. First, a significant increase in cf-DNA was observed in the synovial fluid of animals at 24 h (Fig. 5A). We confirmed the formation of NETs by fluorescence microscopy. The presence of *agg*NETs decorated with H_3_cit were visualized in synovial fluids of ARA suffering heifers (Fig. 5B). Furthermore, the presence of H_3_cit at 24 h was confirmed by Western blot analysis (Fig. 5B). In addition, we observed an increase in serpin B1 at 9 h and 24 h in the synovial fluid of heifers with ARA (Fig. 5C).

### LDH and albumin were increased in the synovial fluid of heifers with ARA

**Fig. 6:**
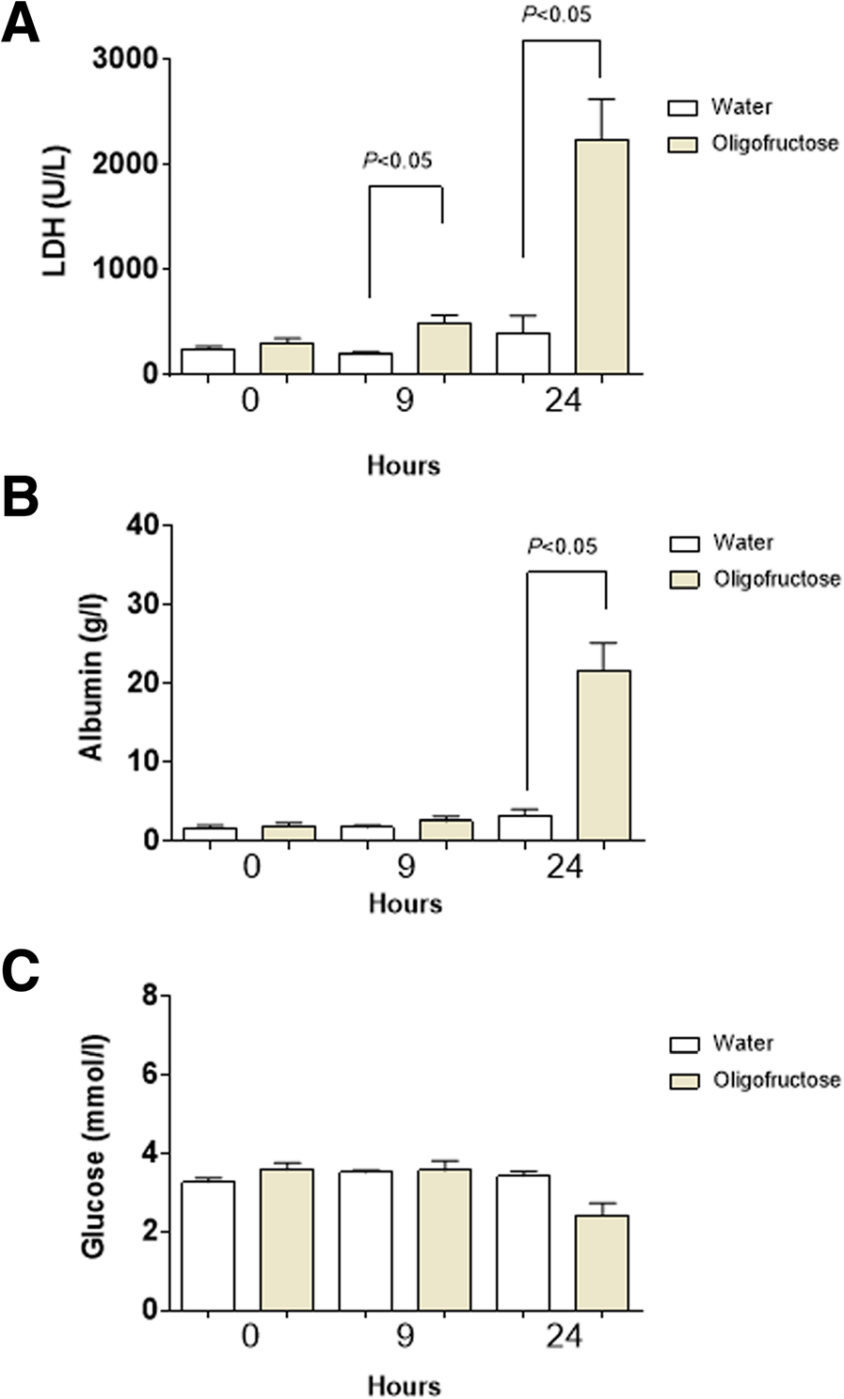
Presence of LDH (**a**), albumin (**b**) and glucose (**c**) in synovial fluid at 0, 9, and 24 h of ARA. Each bar represents the arithmetic mean ± SEM

Some biochemical indicators in synovial fluid have a diagnostic value in joint diseases [35–38]. To assess biochemical changes in the synovial fluid of heifers with ARA we analyzed albumin, LDH and glucose levels. A significant increase in LDH at 9 h and 24 h were observed (Fig. 6a). At 24 h a significant increase in albumin was detected (Fig. 6b). In plasma samples no significant changes for these LDH and albumin parameters were observed (Additional file 1). In contrast, glucose did not change during ARA induction (Fig. 6c).

## Discussion

Ruminal acidosis induces lameness in cattle and previously reported evidence suggests that joint inflammation could be involved [19], however this condition has not been fully characterized. During ARA an increase of pro-inflammatory cytokines IL-6 and IL-1β were observed in the joint at 9 h and 24 h. Lenski and Scherer (2014) [39] recorded an increase in the concentration of IL-6, being the best inflammatory marker for predicting periprosthetic joint infections, however also can be detected in excess in aseptic joint diseases [40–42]. IL-1β is responsible for monocyte survival in the joint [43], and promoting leukocyte attachment and the production of metalloproteinases involved in articular degradation [43] and inflammation [44, 45]. Regardless, in horses synovial fluid IL-1β has no predictive value because of the wide variation obtained in clinically normal joints; which represent the population most likely to have the diagnostic test for evaluating lameness [46]. Therefore, the increase of IL-1β observed in the joints of heifers should be carefully considered as a predictive parameter of lameness in cattle. We detected a high concentration of PGE_2_ in the synovial fluid from heifers with ruminal acidosis; this is a well-known lipid mediator of several joint diseases [47, 48]. Since the increase was observed at 9 h, before neutrophil recruitment, it could be closely related with a possible synoviocyte response in the joint.

Synovitis in heifers with ARA was clinically observed at 24 h and characterized by an increase of total protein concentrations and abundant WBC values [4]. An increase of leukocytes in synovial fluids of cattle suffering arthritis has been previously described [49]. However, an increase of polymorphonuclear neutrophils (PMN) in ruminants has only been associated with infectious arthritis caused by direct inoculation of microorganisms into the joint capsule, extension from periarticular injuries, or by hematogenous spread [49]. Some authors described that the systemic inflammatory response during acute ruminal acidosis could be related to bacterial infection, possibly due to translocation of lipopolysaccharide (LPS) into the peripheral circulation from the rumen [50]. On the contrary, we did not observed changes in proinflammatory parameters in plasma, suggesting non-apparent systemic inflammation. Moreover, other authors demonstrate that the inflammatory processes in the locomotor apparatus during ARA are aseptic [39]. In addition, synovial fluid from animals with ARA, synovitis induced by oligofructose overload, was negative for the presence of bacteria (data not shown); thus being considered by other authors as an aseptic synovitis condition [51]. Therefore, other non-infectious agents/molecules might be involved in the synovium activation during ARA. Until now, the detailed molecular mechanisms to explain on how the pro-inflammatory cascade conveyed from rumen to the joint are still unknown. However, it has been suggested that vasoactive substances produced in the bovine rumen, such as, lactate, histamine, tyramine and tryptamine, might be capable to induce inflammatory processes in the locomotor apparatus of cattle [1].

An increase of ATP concentration in bovine synovial fluid has also been observed in osteoarthritis, sodium urate-induced synovitis in dogs [33], and in arthritis induced in rats [52]. Therefore, extracellular ATP concentrations are nowadays considered as important pro-inflammatory agents [53]. Since ATP was observed at 24 h, this suggests that either neutrophils [54, 55] or synovium [52] can contribute to the release of ATP into the joint.

The increase of MMP-9 activity in synovial fluid from heifers with ARA could be related to the presence of neutrophils in the joint [56], however other cells could also be responsible [57, 58]. In support of this, the synovium as well as leukocytes release MMP-9 contributing to the increase observed in the inflammed joint [58]. The synovial lumen and membrane sections of joints obtained from heifers, both at 24 h and 72 h after oligofructose overload, showed infiltration of neutrophils into the subintima as well as neutrophil accumulation within the synovial lumen [4]. The increase of MMP-9 in the joints of heifers could be closely associated with leukocyte migration and damage observed in tissues adjacent to affected joints.

More importantly, an increase of neutrophils, cf-DNA, and release of *agg*NETs at 24 h in the synovial fluid of heifers with ARA were documented suggesting a potential contribution of *agg*NETs in synovitis. Consistently, in human metabolic gout disease also *agg*NETs were reported to occur in affected joints [59] Peptidylarginine deiminases 4 (PAD4) mediates histone citrullination and promotes NETosis by inducing chromatin decondensation and facilitating the expulsion of chromosomal DNA [60]. In synovial fluid from heifers with ARA, we observed the presence of H3cit decorating *agg*NETs, which have been involved in inflammatory resolution, having the ability to degrade pro-inflammatory cytokines [61]. This suggests that in the joint of heifers with ARA, an *agg*NETs-derived anti-inflammatory mechanism might simultaneously occur, but this speculation request further investigations. In connection with this, we demonstrated the presence of serpin B1 in synovial fluid at 9 h and 24 h, a protein which inhibits neutrophil serine proteases [62] and also able to suppress NETosis [63]. Therefore, an increase in serpin B1 in synovial fluids during acute ruminal acidosis could be related to its ability to resolve inflammatory processes [64] in the joint [65]. Furthermore, other authors using a similar experimental procedure, described that the grade of joint inflammation in dairy heifers is decreased after 24 h of oligofructose overload [4].

During joint inflammation several biochemical markers have been used to characterize and differentiate specific diseases. In the synovial fluid of heifers with ARA a significant increase of albumins and LDH were recorded. Since albumin is only synthesized in the liver [66], its increased concentration in the synovial fluid would indicate an increase in the vascular permeability of affected joints [67]; which could be responsible for the rise in synovial fluid volume observed in the joints of heifers showing ARA-associated clinical manisfestations [4]. In connection with this, the increase of IL-1β, IL-6, and PGE2, all of them considered as potent vascular permeability-inducing agents, might also contribute to explain the increase of albumin and plasma extravasation into joint fluids [67–69].

The increase of LDH enzyme > 2000 U/l in the synovial fluid of heifer with ruminal acidosis was higher than the levels described for clinical intact join in cattle [70] and could be a possible marker of inflammation [36]. The current study found no changes in glucose levels, which agrees with previous findings stating that there is no increase in glucose levels in the synovial fluid during non-infectious joint diseases [39, 71, 72].

## Conclusions

Our data suggest that the joints in heifers with ARA induced by oligofructose overloads resulted not only in an acute inflammatory response with enhanced neutrophils but also in increased of IL-1β-, IL-6-, PGE_2−_, ATP-, and MMP-9-levels within synovial fluids. Simultaneously the presence of *agg*NETs and serpin B1 expression suggest a rather fine-tuning modulation of pro-inflammatory process in affected joints. Aseptic synovitis associated with disorders of reticuloruminal fermentative functions might therefore represent a clinical entity most probably underestimated in cattle industry.

## Additional file

Additional file 1: **Table S1.** Plasmatic pro-inflammatory (IL-1β, IL-6 and PGE_2_) and biochemical (LDH, albumin) parameters of heifers with ARA. (DOCX 14 kb)

## Data Availability

All data generated or analyzed during the study are included in this published article. The datasets used and/or analyzed during the present research project are available from the corresponding author on reasonable request.

## Abbreviations

*agg*NETs: Aggregated NETs
ARA: Acute ruminal acidosis
ATP: Adenosine triphosphate
BW: Body weight
CD11b: Cluster of differentiation molecule 11b
cf-DNA: Cellular free DNA
COX-2: Cyclooxygenase-2
ELISA: Enzyme-linked immunosorbent assay
H3cit: Histone H3 citrullinated
HIF-1α: Hypoxia-inducible factor-1 alpha
ICAM-1: Intercellular adhesion molecule-1
IL-1β: Interleukin-1 beta
IL-6: Interleukin-6
LDH: Lactate dehydrogenase
LPS: Lipopolysaccharide
MMP-9: Matrix metalloproteinase-9
NETs: Neutrophil extracellular traps
PGE_2_: Prostaglandin E_2_
PMN: Polymorphonuclear neutrophils
PVDF: Polyvinylidene difluoride
RT: Room temperature
SDS-PAGE: Sodium dodecyl sulfate–polyacrylamide gel electrophoresis
SEM: Standard error mean
TBS-T: Tris-buffered saline with Tween-20
WBC: white blood cells
