# Supplementary material for: Pro-inflammatory mediators and neutrophils are increased in synovial fluid from heifers with acute ruminal acidosis
Source: BMC Vet Res. 2019 Jul 3;15:225. doi: 10.1186/s12917-019-1974-x (PMC6610826; doi:10.1186/s12917-019-1974-x)
Supplement: Supplementary file 1 — Table S1. Plasmatic pro-inflammatory (IL-1β, IL-6 and PGE2) and biochemical (LDH, albumin) parameters of heifers with ARA. (DOCX 14 kb) [file 12917_2019_1974_MOESM1_ESM.docx]

Table 1: Plasmatic pro-inflammatory (IL-1β, IL-6 and PGE_2_) and biochemical (LDH, albumin) parameters of heifers with ARA.

| Time post-challenge (h) | 0 | | 9 | | 24 | |
| --- | --- | --- | --- | --- | --- | --- |
| Experimental groups | TW | OF | TW | OF | TW | OF |
| IL-1β (pg/ml) | 50.4 ± 0.9^a^ | 52 ± 1.2 ^a^ | 48.7 ± 1.1 ^a^ | 50.4 ± 1.4 ^a^ | 49.8 ± 1.1 ^a^ | 50.4 ± 1.0 ^a^ |
| IL-6 (pg/ml) | 100.5 ± 2.8 ^a^ | 84.6 ± 20.7 ^a^ | 99.8 ± 2.0 ^a^ | 82.8 ± 24.0 ^a^ | 96.7 ± 1.3 ^a^ | 77.2 ± 23.5 ^a^ |
| PGE_2_ (pg/ml) | 124.5 ± 3.0 ^a^ | 100.9 ± 8.7 ^a^ | 124.8 ± 4.6 ^a^ | 99.4 ± 10.4 ^a^ | 122.7 ± 3.4 ^a^ | 98.0 ± 9.5 ^a^ |
| LDH (U/L) | 2055 ± 100.1 ^a^ | 1958 ± 113.6 ^a^ | 1883 ± 143.6 ^a^ | 2018 ± 134.7 ^a^ | 1844 ± 45.2 ^a^ | 1976 ± 155.2 ^a^ |
| Albumin (g/L) | 36.0 ± 1.0 ^a^ | 34.8 ± 0.6 ^a^ | 33.8 ±1.5 ^a^ | 33.6 ± 0.9 ^a^ | 35 ± 1.2 ^a^ | 34.4 ± 1.2 ^a^ |

Each value represent mean ±SEM. The same letter indicates non-significantly differences between tap water (TW) and oligofructose overload (OF) group.
